# Wall thickness–guided persistent atrial fibrillation ablation: have we found the holy grail?

**DOI:** 10.1093/europace/euaf241

**Published:** 2025-09-27

**Authors:** Karan Saraf, Carlos Morillo

**Affiliations:** Libin Cardiovascular Institute, University of Calgary, 3310 Hospital Drive NW, Calgary, Alberta T2N4N1, Canada; Libin Cardiovascular Institute, University of Calgary, 3310 Hospital Drive NW, Calgary, Alberta T2N4N1, Canada; Department of Cardiac Sciences, Libin Cardiovascular Institute, University of Calgary, 3310 Hospital Drive NW, Calgary, Alberta T2N4N1, Canada

**This editorial refers to ‘personalized pulmonary vein isolation guided by left atrial wall thickness for persistent atrial fibrillation ablation: the PeAF-by-LAWT randomized trial’, by Falasconi G. *et al*., https://doi.org/10.1093/europace/euaf163.**

## Personalizing pulmonary vein isolation: beyond fixed ablation targets in persistent atrial fibrillation

Pulmonary vein isolation (PVI) remains the mainstay of ablation for atrial fibrillation (AF), with the mechanistic role of the PVs demonstrated decades ago.^1^ Recurrence frequently results from PV reconnection (PVR), and PV re-isolation improves arrhythmia-free survival.^2,3^ Attempts to ablate targets outside the PVs have not shown significant benefit over PVI alone, but outcomes, particularly in persistent AF (PeAF), remain modest.^4–6^ This underscores the importance of lesion optimization in PVI, as durable isolation yields the best outcomes. How should radiofrequency (RF) be titrated to achieve durable, safe lesions? Advances over recent decades include force-sensing catheters and recognition of the importance of catheter stability, lesion contiguity, and depth. The CLOSE protocol standardized these insights into a reproducible workflow, achieving proven efficiency and safety by setting ablation index (AI) targets by location: ≥ 550 for anterior PV antrum segments and ≥400 for posterior segments, with inter-lesion distance (ILD) ≤ 6 mm, based on the observation that anterior tissue is thicker and the oesophagus lies near the left atrial (LA) posterior wall.^7^ However, emerging imaging studies show marked inter- and intra-patient variability in LA wall thickness (LAWT), from <1 to 5 mm,^8^; therefore fixed AI targets risk under-treating thicker segments (raising PVR risk) and over-treating thinner areas (increasing complication risk, particularly near the oesophagus). Small observational studies tailoring AI targets to LAWT measured by multidetector computed tomography (MDCT) showed feasibility, safety, and efficacy,^9,10^ In the present issue of Europace, Falasconi *et al*.^11^ report on the first testing of this approach in a randomized controlled trial (RCT) comparing it to the ‘standard of care’ CLOSE protocol.

## LAWT-guided vs. CLOSE-guided PVI

The study was a single-blind, multi-centre, non-inferiority RCT randomizing patients undergoing first-time PeAF ablation to either LAWT-guided personalized PVI or standard CLOSE-guided PVI. All patients underwent MDCT integrated into the mapping system. Wide antral circumferential ablation with ILD ≤6 mm was performed in all, but in the LAWT arm, a mandatory right carina line was added, and AI targets adapted to segmental LAWT (from 300 for LAWT <1 mm to 500 for LAWT ≥4 mm); lines were adjusted to avoid unnecessarily thick tissue or oesophageal proximity ≤1 mm, with AI down-titrated to 300 when the latter was not possible. CLOSE-guided ablation used AI ≥550 anteriorly and ≥400 posteriorly. Maximum 35W power was applied in CLOSE and 35–50 W for LAWT.

The primary outcome was freedom from arrhythmia at 12 months with three-monthly ECGs and ambulatory monitors. Anti-arrhythmic medications were discontinued after the blanking period except in those with recurrences. Secondary endpoints included RF time, procedure time, first-pass PVI rate, and fluoroscopy time. Safety endpoints included relevant procedural-adverse events.

One hundred and fifty-six patients were randomized 1:1 between the two strategies; the mean age was 65.6 ± 10.3 years, 69% were male, and other baseline demographics were well-balanced. At 12 months, arrhythmia-free survival was similar between groups (81.3% LAWT vs. 77.6% CLOSE; *P* = 0.50), meeting the non-inferiority margin. LAWT-guided ablation significantly reduced median procedure time (60.5 min [55.0–73.0] vs. 80.0 min [70.0–90.0], *P* < 0.001) and RF time for both left (7.0 min [6.1–8.0] vs. 13.8 min [12.2–15.7]) and right (7.4 min [6.6–8.5] vs. 14.7 min [12.1–16.7]) PV isolation (*P* < 0.001), with no difference between first-pass PVI rate, fluoroscopy time, and distribution of residual gaps. There were no differences in adverse events (1.3% vs. 2.6%, *P* = 0.99).

We applaud the authors for investigating, in a randomized study, a new technique for performing PVI in the context of Persistent AF. Any approach that maintains efficacy and safety while offering personalized therapy instead of a ‘one-size-fits-all’ strategy and that optimizes procedural workflow and efficiency, particularly in the era of faster procedures with pulsed field ablation, should be commended. Advantages include maximizing lesion effectiveness based on objective atrial tissue properties, minimizing unnecessary RF delivery in thin atrial regions, potentially reducing complications such as perforation or atrio-oesophageal fistula, and improving procedure time by up to one quarter, thereby increasing EP lab throughput.

However, there are some limitations to this study. Of note, the population included was primarily composed by early persistent AF highlighting the importance of early intervention given the impressive outcomes that may have been over estimated by the lack of detailed monitoring. These results should not be translated into patients with long-lasting persistent AF. A right-sided carina line was mandatory in LAWT but not CLOSE.^7^ Given that one of the most common areas of PVR is the right carina, this makes the LAWT group a less direct comparison with the CLOSE group when assessing recurrence, as they treated a known problematic area prophylactically in LAWT but not CLOSE.^12^ Similarly, up to 50W power was used with LAWT, but only up to 35W with CLOSE, and whilst AI targets were standardized, this undoubtedly contributed towards shorter RF time with LAWT, rather than personalized AI alone. The study was able to recruit only a small number of patients, and the follow-up period was short. Only seven patients underwent repeat procedures, so it is not known whether recurrence rates in the LAWT group were related to inadequate AI values, one of the main concerns with a novel strategy such as this. One of the main limitations, however, is that whilst it did demonstrate faster procedures and lower RF time, modern practice has in many centres modified the traditional CLOSE protocol, which was originally carried out close to a decade ago, to high power short duration lesions with the same AI targets. Routine use of 50W (and in the case of ‘very high’ power, up to 90W) both anteriorly and posteriorly has significantly reduced contemporary procedure and RF times, and so the main procedural efficiency benefits of LAWT over CLOSE are not as applicable (and overestimate the efficiency of LAWT-guidance) in the modern age of PVI.^13,14^

In summary, the authors deserve praise for conducting an RCT demonstrating a new PVI technique aimed at identifying LA areas where RF delivery can be reduced and the procedure potentially made safer, while maintaining efficacy and improving procedure times, which may render RF PVI more comparable in workflow to pulsed field PVI in the modern era. We believe the study’s limitations preclude its widespread adoption or replacement of CLOSE-guided PVI (particularly when modified with high-power, short-duration lesions), but it represents a valuable step towards personalized ablation, which must remain a future aim in the treatment of persistent AF. This study adds to our quest of finding the ‘holy grail’ strategy for early persistent AF, but our quest is far from over.
